# Nanocellulose Production Using Ionic Liquids with Enzymatic Pretreatment

**DOI:** 10.3390/ma14123264

**Published:** 2021-06-12

**Authors:** Marta Babicka, Magdalena Woźniak, Kinga Szentner, Monika Bartkowiak, Barbara Peplińska, Krzysztof Dwiecki, Sławomir Borysiak, Izabela Ratajczak

**Affiliations:** 1 Department of Chemistry, Faculty of Forestry and Wood Technology, Poznań University of Life Sciences, Wojska Polskiego 75, 60625 Poznań, Poland; marta.babicka21@gmail.com (M.B.); magdalena.wozniak@up.poznan.pl (M.W.); kinga.szentner@up.poznan.pl (K.S.); 2 Department of Chemical Wood Technology, Faculty of Forestry and Wood Technology, Poznań University of Life Sciences, Wojska Polskiego 38/42, 60627 Poznań, Poland; monika.bartkowiak@up.poznan.pl; 3 NanoBioMedical Centre, Adam Mickiewicz University, Wszechnicy Piastowskiej 3, 61614 Poznań, Poland; barp@amu.edu.pl; 4 Department of Food Biochemistry and Analysis, Faculty of Food Science and Nutrition, Poznań University of Life Sciences, Mazowiecka 48, 60623 Poznań, Poland; krzysztof.dwiecki@up.poznan.pl; 5 Institute of Chemical Technology and Engineering, Poznan University of Technology, Berdychowo 4, 60965 Poznań, Poland; slawomir.borysiak@put.poznan.pl

**Keywords:** nanocellulose, ionic liquids, *Trichoderma reesei*, enzymatic hydrolysis

## Abstract

Nanocellulose has gained increasing attention during the past decade, which is related to its unique properties and wide application. In this paper, nanocellulose samples were produced via hydrolysis with ionic liquids (1-ethyl-3-methylimidazole acetate (EmimOAc) and 1-allyl-3-methylimidazolium chloride (AmimCl)) from microcrystalline celluloses (Avicel and Whatman) subjected to enzymatic pretreatment. The obtained material was characterized using Fourier transform infrared spectroscopy (FTIR), X-ray diffraction (XRD), dynamic light scattering (DLS), scanning electron microscopy (SEM), and thermogravimetric analysis (TG). The results showed that the nanocellulose had a regular and spherical structure with diameters of 30–40 nm and exhibited lower crystallinity and thermal stability than the material obtained after hydrolysis with *Trichoderma reesei* enzymes. However, the enzyme-pretreated Avicel had a particle size of about 200 nm and a cellulose II structure. A two-step process involving enzyme pretreatment and hydrolysis with ionic liquids resulted in the production of nanocellulose. Moreover, the particle size of nanocellulose and its structure depend on the ionic liquid used.

## 1. Introduction

Nanocellulose has gained increasing attention during the past decade, as confirmed by the number of patents and scientific papers related to its properties, production methods, and potential applications [1,2]. Cellulose nanocrystals have found applications in various fields, for example as food packaging, biodegradable polymers, biomedical utilization (including drug delivery, substituted implants, biocatalyst or tissue regeneration), wood adhesives, or fillers and additives to nanocomposites [2,3,4,5,6,7,8,9,10]. Nanocellulose-based polymer composites have potential applications as adsorptive, filtering, and decontaminating materials (including for water treatment, air purification, or microbe and viral decontamination), as well as materials in binders, separators, and electrodes of energy conservation devices and energy capture devices, e.g., as CO_2_ separators [8,11,12,13,14,15]. The wide application of nanocellulose is connected with its unique properties, such as its high surface area, light weight, low density, biodegradability, biocompatibility, and outstanding strength properties [16,17,18].

Numerous methods are applied to produce cellulose with nanometric dimensions from different lignocellulose materials, including chemical (e.g., with the use of acids and bases) and physical (e.g., grinding, grating, or with the use of high-power lasers) methods [19,20,21]. A common method of nanocellulose production is acid hydrolysis, along with its modifications, whereby sulfuric, hydrobromic, and hydrochloride acids are usually used in the process of cellulose hydrolysis [8,22,23]. However, acid hydrolysis is not considered an environmentally friendly method due to the use of large amounts of solvents, which generate a considerable volume of sewage that requires treatment and contributes to the corrosion of reactors. Moreover, acid hydrolysis is characterized by the low efficiency of the nanocellulose production and the formation of cellulose nanocrystals with reduced thermal stability [24,25,26].

An eco-friendly alternative method of nanocellulose production is the application of ionic liquids or enzymes, since these methods do not generate hazardous waste, as is the case with acid hydrolysis. Different classes of enzymes have been applied in nanocellulose preparations, including cellulases, xylanases, and lytic polysaccharide monooxygenases [27]. However, cellulases, which are produced by cellulolytic organisms, including fungal species such as *Aspergillus*, *Trichoderma,* or *Clostridium*, are the most commonly used in preparation of nanocellulose [27,28]. It is generally recognized that complete hydrolysis of cellulose to glucose requires a synergistic action of at least two of the three groups into which cellulases are divided, namely endoglucanases, exoglucanases, and cellobiohydrolases [1,29]. However, for the production of nanocellulose, endoglucanases are of greatest interest due to their action on amorphous cellulose [30]. It should also be emphasized that the efficiency of the enzymatic hydrolysis process depends on the types of cellulolytic enzymes that determine the sizes of nanometric particles, as well as their polydispersion [28]. According to data from the literature, enzymes are used in the extraction of nanometric cellulose, both alone or combined with chemical or mechanical methods [22,27,31,32,33,34,35,36,37,38]. Moreover, the efficiency of enzymatic cellulose hydrolysis depends on many other factors, such as the crystallinity, average molecular weight, polymorphism, and lignin or hemicellulose contamination [39,40,41].

Ionic liquids, often referred to as green solvents, have been used in the production of nanocellulose [42,43,44,45]. Ionic liquids (ILs) are generally defined as salts that melt below 100 °C and are completely composed of ions. Interestingly, ILs have many attractive properties such as chemical and thermal stability, low melting points, non-volatility and non-flammability, low vapor pressures, and recyclability [46,47]. Various types of ionic liquids are used in nanocellulose hydrolysis, including 1-butyl-3-methylimidazolium hydrogen sulfate, 1-butyl-3-methylimidazolium chloride, and 1-ethyl-3-methylimidazole chloride [23,48,49,50,51,52,53]. The production of nanocellulose using ionic liquids has numerous benefits, including the potential to use atmospheric pressure, small amounts of solvents, the potential for regeneration of ionic liquids, and working with an odorless and relatively safe solvent. On the other hand, this method also has disadvantages, which include the relatively high costs of ionic liquids and the unsatisfactory efficiency of the extraction process [51,54,55,56,57,58,59].

Combinations of methods have been used to increase the efficiency of the nanocellulose production process. The aim of pretreatment is to bring the cellulose polymers to an appropriate form that will increase the efficiency of the subsequent process of nanocellulose production itself. Pretreatment can be a physical or chemical process; most often it is associated with reducing the particle size of the cellulose, increasing the porosity and surface area, or reducing the crystallinity [60,61]. Prior to enzymatic hydrolysis reactions, lignocellulosic materials are pretreated using various methods, including milling, swelling treatment, steam explosion, sonification, or treatment with a aqueous sodium hydroxide solution and ionic liquids [22,31,37,62,63].

In our paper, we use enzymatic hydrolysis of cellulosic materials as a preliminary stage of nanocellulose preparation with the application of ionic liquids. To our knowledge, this is the first time that enzymatic hydrolysis has been combined with treatment with ionic liquids to obtain nanocellulose, where enzymatic hydrolysis is the pretreatment step. Therefore, the aim of the study is to produce nanometric cellulose by pretreatment with the cellulolytic enzyme from *Trichoderma reesei*, followed by treatment with two ionic liquids: 1-ethyl-3-methylimidazole acetate (EmimOAc) and 1-allyl-3-methylimidazolium chloride (AmimCl). The obtained material is characterized using Fourier transform infrared spectroscopy (FTIR), X-ray diffraction (XRD), dynamic light scattering (DLS), thermogravimetric analysis (TG), and scanning electron microscope (SEM).

## 2. Materials and Methods

### 2.1. Materials

Microcrystalline cellulose: Avicel PH-101 and Whatman cellulose filter paper No. 1 were purchased from Sigma Aldrich Chemie GmbH (Darmstadt, Germany). The cellulolytic enzyme from the microscopic fungus *Trichoderma reesei* ATCC 26921 with an activity of 700 units/g and the ionic liquids 1-allyl-3-methylimidazolium chloride (≥97.0%) and 1-ethyl-3-methylimidazolium acetate (≥95.0%) were also purchased from Sigma Aldrich Chemie GmbH (Darmstadt, Germany).

### 2.2. Pretreatment of Cellulose with Cellulolytic Enzyme

The cellulose material (Avicel and Whatman) was added to a citrate buffer (50 mM, pH = 4.8) at a ratio of 50:1 (mg/mL) and was incubated for 30 min at 50 °C with a shaking speed of 150 rpm/min (Incubated Shaker, Lab Companion, JeioTech, Korea). Afterwards, the cellulolytic enzyme diluted in the citrate buffer (1:50 by volume) was added to the cellulose material at a ratio of 1:2 by volume. The mixture was incubated at 50 °C with a shaking speed of 150 rpm for 30 min. The reaction was stopped by boiling the sample for 5 min. Next, the samples were centrifuged at 1000 rpm/min for 15 min (Universal 320, Andreas Hettich GmbH and Co. KG, Tuttlingen, Germany) and washed with deionized water. The solid cellulose residue was dried in a laboratory dryer (Pol-Eko-Aparatura, Wodzisław Śląski, Poland) and used for further analysis.

### 2.3. Preparation of Nanocellulose by Ionic Liquids

The cellulose material (Avicel and Whatman) after pretreatment with the *Trichoderma reesei* enzyme was mixed with ionic liquids ((EmimOAc) and (AmimCl)) at a ratio of 1:5 by weight. The reactions were run until the material had a homogeneous mix (~15 min) at 80 °C, under intense stirring using a heating mantle with magnetic stirring (ChemLand, Stargard, Poland). The reaction was carried out without solvent. The reaction was stopped by adding 15 mL of an acetone and water mixture (1:1) to the reaction mixture. The products of reactions were washed with the acetone and water mixture, filtered, and dried initially at room temperature and finally over P_2_O_5_ (Sigma Aldrich Chemie GmbH, Darmstadt, Germany).

### 2.4. Methods

#### 2.4.1. FTIR Spectroscopy

Fourier transform infrared spectroscopy was used to characterize the obtained materials and determine their chemical structure. All samples (1 mg) were mixed with KBr (200 mg) (Sigma Aldrich Chemie GmbH, Darmstadt, Germany) and analyzed in the pastille form. Spectra were recorded in the range of 4000–500 cm^−1^, with a resolution of 2 cm^−1^, and 16 scans were recorded on a Nicolet iS5 spectrophotometer (Thermo Fisher Scientific, Waltham, MA, USA).

#### 2.4.2. XRD Analysis

The supermolecular structures of cellulose samples after enzymatic hydrolysis and enzyme-pretreated cellulose treated with ionic liquids were analyzed using the X-ray diffraction (XRD) analysis. The samples were determined using a TUR M-62 X-ray diffractometer (Carl Zeiss AG, Jena, Germany) with a copper anode. The wavelength of the Cu $K_{\alpha }$ radiation source was 1.5418 Å and the spectra were obtained at 30 mA with an accelerating voltage of 40 kV. The diffraction pattern was recorded between 5 and 30° (2θ-angle range) in the step of 0.04°/3 s. Deconvolution of peaks was performed using the method proposed by Hindeleh and Johnson [64] and improved and programmed by Rabiej [65]. After separation of XRD lines, the degrees of crystallinity (X_c_) of cellulose samples were determined by comparing the areas under crystalline peaks and the amorphous curve.

#### 2.4.3. DLS Analysis

The particle sizes (expressed as the hydrodynamic diameter) of cellulose samples were determined using the DLS method, using a Zetasizer Nano ZS-90 instrument (Malvern, UK). Before analysis, the tested materials were mixed (2 mg) with deionized water (5 mL) and treated using an ultrasound bath (Polsonic, Warsaw, Poland) for 25 min.

#### 2.4.4. SEM Analysis

The surface morphologies of micro- and nanocrystalline cellulose were examined using the SEM method. Images were taken with the use of a JEOL JSM-7001F TTLS scanning electron microscope (JEOL Ltd., Tokyo, Japan) by applying the accelerating voltage of 5 kV and a secondary electron (SEI) detector. The samples were placed on a carbon tape and investigated without coating.

#### 2.4.5. TG Analysis

Thermogravimetric analysis was performed on the Netzsch STA 449 F5 Jupiter apparatus (Erich NETZSCH GmbH and Co. Holding KG, Selb, Germany). The tested cellulose samples (15 ± 1 mg) were heated at the rate of 10 °C/min to the assumed temperature of 600 °C. The analyses were performed in an atmosphere of helium flowing through the furnace space at a rate of 15 mL/min. Thermogravimetric curves (TG) and differential thermogravimetric curves (DTG) were recorded on the thermograms. The former illustrate the dependence of the change in mass (mass loss) on temperature, while the latter illustrate the rate of this change.

## 3. Results and Discussion

### 3.1. FTIR Analysis

**Figure 1:**
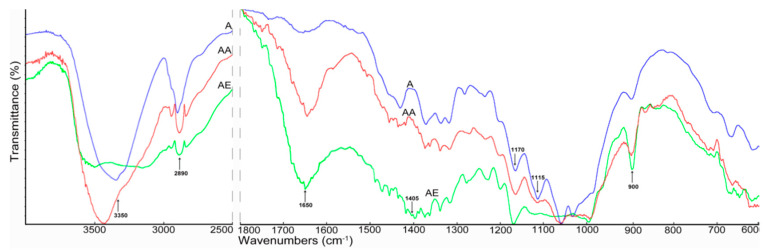
FTIR spectra of (A) enzyme-pretreated Avicel cellulose, (AA) enzyme-pretreated Avicel cellulose treated with AmimCl, and (AE) enzyme-pretreated Avicel cellulose treated with EmimOAc.

**Figure 2:**
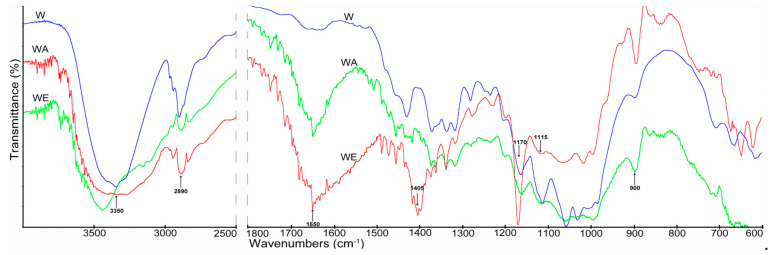
FTIR spectra of (W) enzyme-pretreated Whatman cellulose, (WA) enzyme-pretreated Whatman cellulose treated with AmimCl, and (WE) enzyme-pretreated Whatman cellulose treated with EmimOAc.

In the first stage of the research, the chemical structures of the cellulose samples were determined using Fourier transform infrared spectroscopy (FTIR). The FTIR spectra of the cellulose materials after enzymatic hydrolysis and the materials obtained after the two-step nanocellulose production process (pretreatment with the enzyme and treatment with ionic liquids) are shown in Figure 1 and Figure 2.

**Figure 3:**
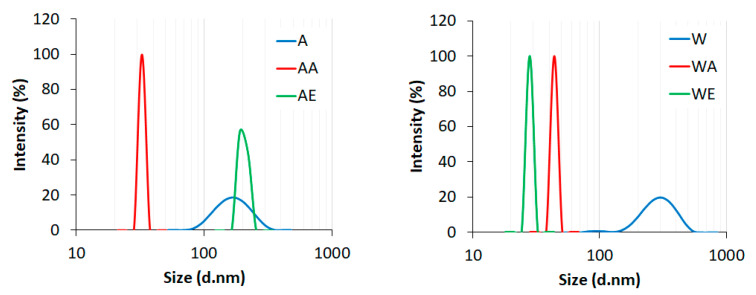
The average particle sizes of (A) enzyme-pretreated Avicel cellulose, (AA) enzyme-pretreated Avicel cellulose treated with AmimCl, (AE) enzyme-pretreated Avicel cellulose treated with EmimOAc, (W) enzyme-pretreated Whatman cellulose, (WA) enzyme-pretreated Whatman cellulose treated with AmimCl, and (WE) enzyme-pretreated Whatman cellulose treated with EmimOAc.

All cellulose samples (Avicel and Whatman), including those after enzyme hydrolysis and enzyme-pretreated cellulose hydrolyzed with ionic liquids, presented a broad band in the region of 3350–3480 cm^−1^, which can be attributed to the hydrogen bond O–H stretching vibrations and flexural vibration of intra- and intermolecular hydrogen bonds [47,66]. The changes in intensity of peaks in this region observed in the spectra of cellulose treated with ionic liquids compared to the spectra of samples after enzyme hydrolysis may have been connected to changes in the number and strength of hydrogen bonds. The band at 2900 cm^−1^ attributed to C–H stretching vibrations was observed in the spectra of all cellulose samples; however, the samples treated with ionic liquids show higher intensities [23,47]. The band at 1650 cm^−1^ visible in the spectra of cellulose treated with ionic liquids was connected with –OH bending of absorbed water [23,47,67]. The intensity of the band at 1650 cm^−1^ was higher for cellulose samples treated with ionic liquids than for samples treated with the cellulosic enzyme. This was associated with the larger surface area of cellulose particles with smaller dimensions (Figure 3), and thus the greater ability to adsorb moisture. Moreover, significant changes were found within the vibration bands of the amorphous region at 900 cm^−1^ and crystalline regions at 1405 cm^−1^. The changes indicated a decrease in cellulose crystallinity after the applied material treatment. These observations were confirmed by the XRD analysis (Table 1). The peaks at 1170 cm^−1^ and 900 cm^−1^ were connected with C–O stretching or O–H bending and the glycosidic C_1_–H deformation mode, respectively [48]. In turn, the bands at 1405 cm^−1^ and 1115 cm^−1^ were attributed to the C–H deformation (asymmetric) and O–H association band in cellulose, respectively [68]. Moreover, in the spectra of treated cellulose samples, especially samples hydrolyzed with ionic liquids, peaks ranging from 1100 to 550 cm^−1^ were observed, indicating twisting, wagging, and deformation modes of anhydro-glucopyranose, which represent the characteristic pattern of β-glucosidic linkages [23,47].

### 3.2. DLS Analysis

The average particle sizes (hydrodynamic diameter) of the enzyme-pretreated cellulose and nanocellulose samples obtained after the two-step production process as assessed by dynamic light scattering (DLS) are presented in Figure 3.

The enzymatic hydrolysis of cellulosic materials (Avicel and Whatman) resulted in a decrease of the average particle size compared to the starting material, which was micrometric in size. The initial particle size range of Avicel cellulose was 1300–4800 nm, while Whatman cellulose had a particle size range of 700–6400 nm. After enzyme pretreatment, the average size of Avicel cellulose particles was below 200 nm, while the particle size of Whatman cellulose was about 300 nm. However, both of the peaks were wide, indicating high particle size dispersion. According to the data from the literature, the average particle size for cellulose hydrolyzed with the cellulase enzyme was 0.526 µm; however, 50% of the particles were smaller than this [66]. The average size range of cellulose nanocrystals obtained via endoglucanase enzyme hydrolysis with two heating models (conventional and microwave-coupled with ultrasonication) was 100 nm to 3.5 µm [69]. In turn, the particle size of nanocellulose prepared through enzymatic hydrolysis with three different pretreatments (ultrasonic treatment, treatment with NaOH, and treatment with DMSO) depended on the pretreatment type. The average size of nanocellulose with ultrasonic pretreatment was 5–6 nm, for nanocellulose prepared by DMSO pretreatment was about 250 nm, while nanocellulose obtained with NaOH pretreatment contained two particle sizes—one type measuring 25 nm and the other measuring 250 nm, which were the aggregates [67].

The results presented in Figure 3 indicate a notable shift in the cellulose particle size after treatment with both ionic liquids (except for Avicel cellulose treated with EmimOAc) as compared to the enzyme-pretreated cellulose. In addition, all nanocellulose samples were characterized by much lower particle size dispersions than the samples after enzymatic hydrolysis, confirming the narrow and well-defined peaks presented in Figure 3. The average particle size for enzyme-pretreated Avicel cellulose obtained after hydrolysis with AmimCl (AA) and enzyme-pretreated Whatman cellulose obtained after hydrolysis with EmimOAc (WE) was around 30 nm, while that of enzyme-pretreated Whatman cellulose obtained after hydrolysis with AmimCl (WA) was around 40 nm. In turn, the nanocellulose obtained by Avicel cellulose hydrolysis with EmimOAc (AE) was characterized by the greater average size of particles (around 200 nm) compared to the other nanocellulose samples obtained after hydrolysis with ionic liquids. To sum up, the results of the DLS analysis confirmed that the efficiency of nanocellulose production with enzyme pretreatment is influenced by the type of the initial cellulosic material, as well as the type of ionic liquid used.

### 3.3. SEM Analysis

**Figure 4:**
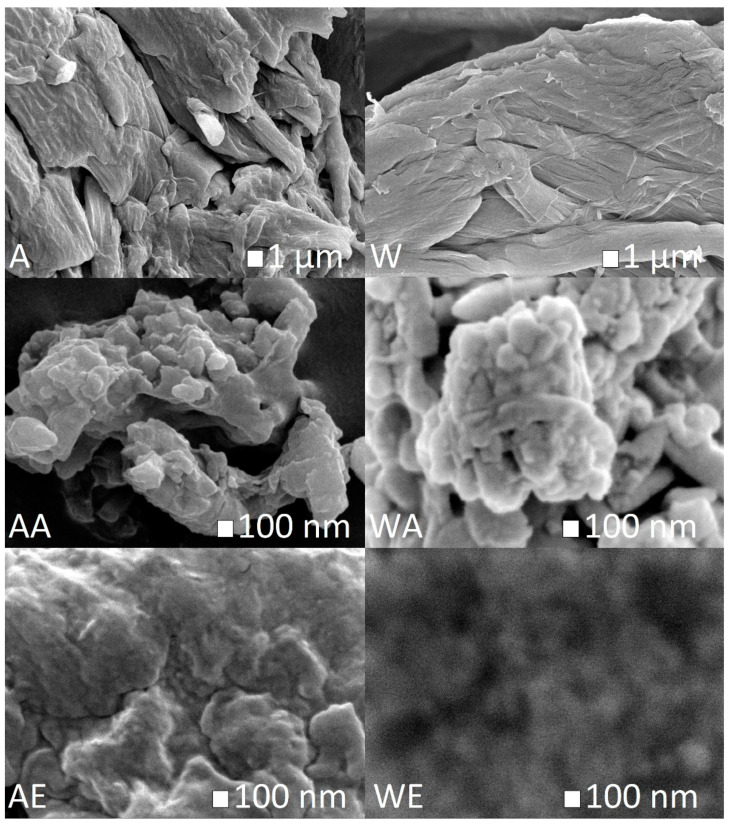
SEM images of (**A**) enzyme-pretreated Avicel cellulose, (**AA**) enzyme-pretreated Avicel cellulose treated with AmimCl, (**AE**) enzyme-pretreated Avicel cellulose treated with EmimOAc, (**W**) enzyme-pretreated Whatman cellulose, (**WA**) enzyme-pretreated Whatman cellulose treated with AmimCl, and (**WE**) enzyme-pretreated Whatman cellulose treated with EmimOAc.

The morphologies of the cellulosic materials after enzymatic pretreatment followed by hydrolysis with ionic liquids were examined by SEM analysis. The SEM images are shown in Figure 4.

Figure 4 shows that hydrolysis of enzyme-pretreated cellulose (Avicel and Whatman) with both ionic liquids caused changes in the material structure and a reduction of its diameter. The cellulose material after treatment with ionic liquids had a more regular and spherical structure than cellulose hydrolyzed with the *Trichoderma reesei* enzyme. The spherical structure of obtained nanocellulose is in contrast to the results of other research, where nanocelluloses produced by hydrolysis with an ionic liquid had needle- or rod-like morphologies [23,44,47,70].

### 3.4. XRD Analysis

**Figure 5:**
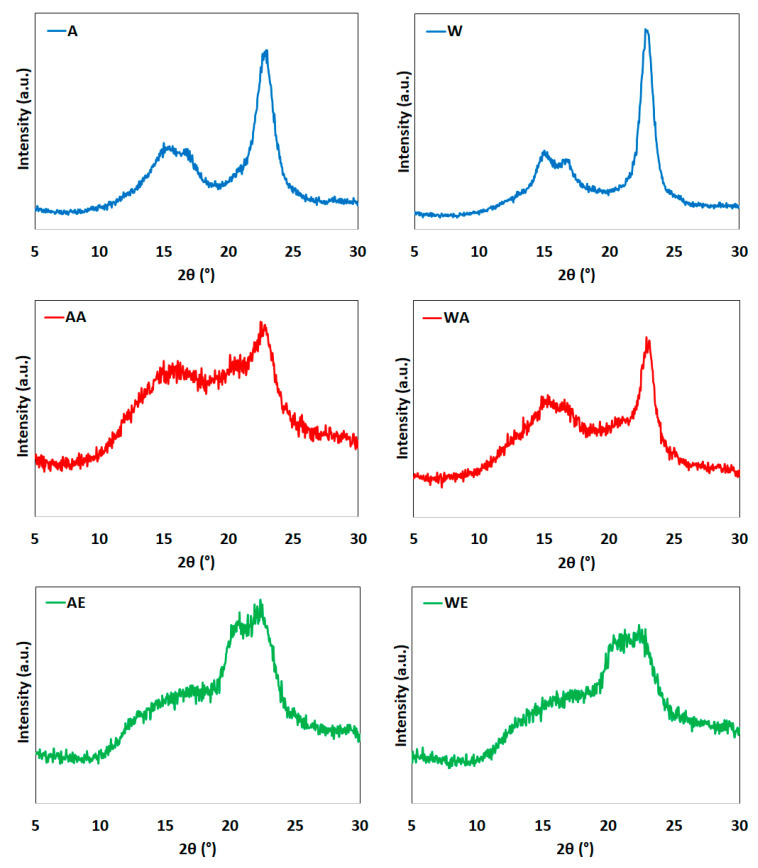
XRD patterns of (**A**) enzyme-pretreated Avicel cellulose, (**AA**) enzyme-pretreated Avicel cellulose treated with AmimCl, (**AE**) enzyme-pretreated Avicel cellulose treated with EmimOAc, (**W**) enzyme-pretreated Whatman cellulose, (**WA**) enzyme-pretreated Whatman cellulose treated with AmimCl, and (**WE**) enzyme-pretreated Whatman cellulose treated with EmimOAc.

The diffraction profiles of enzyme-pretreated cellulose and cellulose after treatment with ionic liquids are shown in Figure 5.

The diffractograms of cellulose after enzymatic hydrolysis (Avicel and Whatman) showed peaks characteristic of cellulose I at 2θ = 15° (plane 1–10), 17° (plane 110), and 22.7° (plane 200) [71]. Additionally, these maxima were observed for the enzyme-pretreated cellulose samples treated with AmimCl, although the intensity was much lower.

Unexpectedly, in the case of enzyme-pretreated cellulose treated with EmimOAc, disappearance of the maximum from cellulose I was observed, along with the formation of two maxima at the diffraction angles of 20° and 22°, the peaks of which came from the lattice planes at (110) and (020), respectively. This indicates the formation of cellulose II and proves that the use of the 1-ethyl-3-methylimidazolium acetate solution resulted in the conversion of polymorphic cellulose I into cellulose II. Data from the literature indicated that the conversion of cellulose I into cellulose II is the result of a mercerization process run in the presence of alkali [72,73]. Additionally, Cheng et al. [74] noted that the use of 1-ethyl-3-methylimidazolium acetate for modification of cellulose, switchgrass (*Panicum virgatum*), pine (*Pinus radiata*), and eucalyptus (*Eucalyptus globulus*) affects the formation of cellulose II. The transformation of cellulose I into cellulose II was also noted by Li et al. [75] and by Wang et al. [76] for another ionic liquid, namely 1-butyl-3-methylimidazolium chloride.

Table 1 presents the crystallinity index values for the cellulose samples.

The values of the crystallinity index indicated that enzymatic hydrolysis affected the degree of crystallinity of the raw cellulose material. The crystallinity index values for enzyme-pretreated cellulose were 60% for Avicel and 63% for Whatman types compared to 61% and 75% for untreated raw Avicel and Whatman cellulose, respectively [44,77]. However, significant changes in the degree of crystallinity were noted for the cellulose samples obtained after hydrolysis with ionic liquids. The calculated crystallinity index values for Avicel cellulose after the reaction with ionic liquids were 24% (after hydrolysis with AmimCl) and 36% (after hydrolysis with EmimOAc). The crystallinity values for Whatman cellulose after the reaction with ionic liquids were 33% (after hydrolysis with AmimCl) and 39% (after hydrolysis with EmimOAc). Among all of the cellulose samples, the nanocellulose sample with the smallest particle size (AA) (Figure 3) had the lowest crystallinity. This indicates that the cellulose chains were broken during the treatment process with ionic liquids, with a consequent reduction in the degree of crystallinity. The reaction of enzyme-pretreated cellulose with AmimCl caused decreases of the crystallinity index by about 60% for Avicel cellulose and 48% for Whatman cellulose compared to the enzyme-pretreated material. Such reductions in the degree of crystallinity for cellulose treated with ionic liquids were previously reported by other authors [44,70,76]. The degree of crystallinity of eucalyptus pulp amounting to 70% was reduced to 36% by treatment with an ionic liquid, namely BmimCl [76]. The degrees of crystallinity for nanocelluloses obtained from cotton and microcrystalline cellulose treated with BmimCl were 52% and 62% compared to the values of 77% and 80% for the native material, respectively [50]. The degree of crystallinity for the nanocellulose obtained with EmimOAc (33%) was lower than the degree of crystallinity for the nanocellulose obtained in our previous work, where EmimCl (47%) was used [44].

According to the literature date, the anion of the ionic liquid plays an important role in the dissolution and hydrolysis process of cellulose, which forms hydrogen bonds with -OH groups of cellulose. The hydrogen bonds of the cations of ionic liquid are most likely formed mainly between the H1 proton of the imidazolium ring and the C6 and C3 carbons of the cellulose chain [78,79]. The research indicated that the acetate anion forms strong hydrogen bonds, as opposed to the weakly basic chloride anion, which forms weak hydrogen bonds with cellulose [24,53,78].

### 3.5. TG Analysis

**Figure 6:**
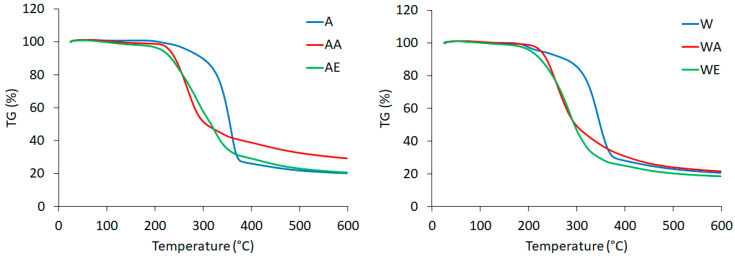
TG curves of (A) enzyme-pretreated Avicel cellulose, (AA) enzyme-pretreated Avicel cellulose treated with AmimCl, (AE) enzyme-pretreated Avicel cellulose treated with EmimOAc, (W) enzyme-pretreated Whatman cellulose, (WA) enzyme-pretreated Whatman cellulose treated with AmimCl, and (WE) enzyme-pretreated Whatman cellulose treated with EmimOAc.

**Figure 7:**
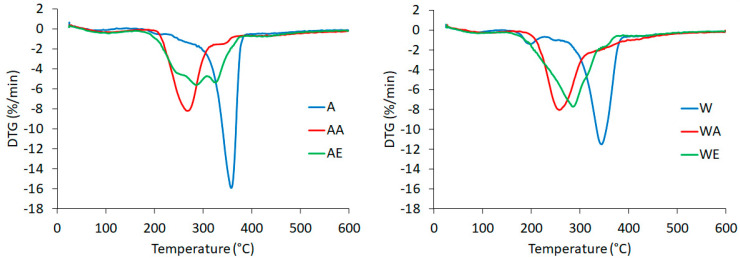
DTG curves of (A) enzyme-pretreated Avicel cellulose, (AA) enzyme-pretreated Avicel cellulose treated with AmimCl, (AE) enzyme-pretreated Avicel cellulose treated with EmimOAc, (W) enzyme-pretreated Whatman cellulose, (WA) enzyme-pretreated Whatman cellulose treated with AmimCl, and (WE) enzyme-pretreated Whatman cellulose treated with EmimOAc.

The thermogravimetric curve (TG) and its derivative curve (DTG) for enzyme-pretreated celluloses (in Avicel and Whatman forms) hydrolyzed with ionic liquids are shown in Figure 6 and Figure 7, respectively.

Knowledge of the thermostability of nanocellulose is important, e.g., when this material is used as a filler for a biopolymer, where elevated temperatures are required for their production [27,63,80]. Raw cellulose is a material with moderate thermal properties, with decomposition temperature ranging between 315 and 400 °C [81]. The TG and DTG curves for enzyme hydrolyzed cellulose differed from those for enzyme-pretreated cellulose after the reaction with both ionic liquids, which indicated that hydrolysis with ionic liquids results in changes in the characteristic degradation temperatures. All of the tested samples initially showed slight losses of weight at temperatures below 100 °C, which were related to water evaporation [47]. The presence of hydrogen bonds in the tested cellulose materials was confirmed by FTIR analysis (Figure 1). In the curves for all samples, except for the EmimOAc-treated cellulose, a single significant decomposition process was observed, corresponding to the degradation processes, such as dehydration, depolymerization, and degradation of the glycosyl rings, followed by the formation of a charred residue [66]. The DTG curve for the enzyme-pretreated Avicel cellulose hydrolyzed with EmimOAc showed a different course of thermal decomposition (two peaks on the DTG curve) and different thermal characteristics (thermal properties) compared to the other cellulose samples. The thermal behavior of this cellulose sample can be explained by the particle size (around 200 nm), which was greater than that of the other nanocellulose samples. This may have caused thermal degradation to take place in multiple stages (two peaks) and to be slower (the maximum rates of decomposition are lower compared to the other tested samples, as shown on the y axes (%/min) for the DTG curves). The onset temperature of degradation (T_onset_) and the maximum decomposition temperature (T_max_) for the enzyme-pretreated Avicel cellulose were 300 and 358 °C, respectively. In turn, decomposition of the enzyme-pretreated Whatman cellulose took place within a temperature range of 293–379 °C.

The degradation behaviors of the enzyme-pretreated celluloses hydrolyzed with ionic liquids presented differences from that of the cellulose treated only with the enzyme, implying that the degradation started at lower temperatures of 211 °C (Avicel) and 188 °C (Whatman) for the cellulose treated with AmimCl and 194 °C (Avicel) and 200 °C (Whatman) for the cellulose treated with EmimOAc. The beginning of the decomposition for the enzyme-pretreated cellulose after the reaction with ionic liquids at lower temperatures was connected with the lower crystallinity index values of celluloses treated with AmimCl and EmimOAc. The high surface area of cellulose nanoparticles also reduces their thermal properties because of the greater surface area exposed to high temperatures [66]. The nanocelluloses obtained using different methods were characterized by varying thermal stability. The onset temperatures for the enzyme-pretreated cellulose after the reaction with both ionic liquids were lower than those for the nanocelluloses obtained from wood (280 °C), maize husk (291 °C), and sugar cane (319 °C), as described in a paper by Onkarappa et al. [68]. In turn, decomposition of cellulose nanocrystals obtained via hydrolysis with sulfuric acid started at around 150 °C, as described by Lu and Hsieh [82]. The nanocellulose obtained by hydrolysis with BmimCl showed lower thermal stability with a decomposition temperature of 238 °C as compared to 288 °C for raw cellulose [75]. Lower thermal stability was also exhibited by the nanocellulose produced by hydrolysis with BmimHSO_4_ as compared to native microcrystalline cellulose [47].

## 4. Conclusions

The present study has demonstrated that nanocellulose could be produced through hydrolysis with ionic liquids from enzyme-pretreated microcrystalline cellulose. In this study, ionic liquids (1-ethyl-3-methylimidazole acetate (EmimOAc) and 1-allyl-3-methylimidazolium chloride (AmimCl)) were used. The ionic liquid treatment of the cellulosic material (Avicel and Whatman) obtained after hydrolysis with enzymes from *Trichoderma reesei* resulted in a decrease of the average particle size compared to the material after enzymatic hydrolysis. The nanocellulose samples were found to have a regular and spherical structure with diameters of about 30–40 nm. The exception was cellulose obtained from the enzyme-pretreated Avicel cellulose via hydrolysis with EmimOAc, which had a particle size of about 200 nm. The basic cellulose I structure was preserved in cellulose obtained after hydrolysis with the *Trichoderma reesei* enzyme and nanocellulose obtained through AmimCl treatment. In the case of enzyme-pretreated cellulose treated with EmimOAc, the transformation to cellulose II occurred. All nanocellulose samples showed decreases in crystallinity index values compared to the material after enzymatic hydrolysis. Moreover, treatment with ionic liquids changed the thermal properties of nanocellulose, resulting in decreases in their thermal stability.

Overall, the two-step process involving enzyme pretreatment and hydrolysis with ionic liquids resulted in the production of nanocellulose. The presented results indicate that the particle size of nanocellulose and its structure depend on the ionic liquid used.

## Figures and Tables

**Table 1:** The crystallinity index values for the cellulose samples.

Samples	The Crystallinity Index (%)	Samples	The Crystallinity Index (%)
A	60	W	63
AA	24	WA	33
AE	36	WE	39

## Data Availability

The data reported in this study are available from the authors upon request.
